# Cervical cancer awareness among women recently diagnosed with cervical cancer in South Africa and Zimbabwe

**DOI:** 10.3332/ecancer.2025.2018

**Published:** 2025-10-17

**Authors:** Sudarshan Govender, Tamsin K Phillips, Fiona M Walter, Sarah Day, Bothwell Guzha, Suzanne E Scott, Zvavahera M Chirenje, John E Ataguba, Nomonde Mbatani, Nazia Fakie, Jennifer Moodley

**Affiliations:** 1 Division of Public Health Medicine, School of Public Health, Faculty of Health Sciences, University of Cape Town, Cape Town 7925, South Africa; 2 Division of Epidemiology and Biostatistics, School of Public Health, Faculty of Health Sciences, University of Cape Town, Cape Town 7925, South Africa; 3 Barts and The London School of Medicine and Dentistry, Wolfson Institute of Population Health, Queen Mary University of London, London EC1M 6BQ, UK; 4 Department of Obstetrics and Gynaecology, Faculty of Medicine and Health Sciences, University of Zimbabwe, Box A178, Harare, Zimbabwe; 5 Bixby Centre for Global Reproductive Health, University of California, San Francisco, CA 94158, USA; 6 Health Economics Laboratory, College of Community and Global Health, Rady Faculty of Health Sciences, University of Manitoba, Winnipeg, MB R3E 3P5, Canada; 7 Health Economics Unit, School of Public Health, Faculty of Health Sciences, University of Cape Town, Cape Town 7925, South Africa; 8 School of Health Systems and Public Health, University of Pretoria, Pretoria 0002, South Africa; 9 Partnership for Economic Policy, Nairobi 00100, Kenya; 10 Department of Obstetrics and Gynaecology, Faculty of Health Sciences, University of Cape Town, Cape Town 7925, South Africa; 11 Department of Radiation Oncology, Faculty of Health Sciences, University of Cape Town, Cape Town 7925, South Africa; a https://orcid.org/0000-0003-1565-0703; b https://orcid.org/0000-0003-4554-2922; c https://orcid.org/0000-0002-7191-6476; d https://orcid.org/0000-0003-2165-3580; e https://orcid.org/0000-0002-3434-1677; f https://orcid.org/0000-0001-5536-9612; g https://orcid.org/0000-0003-3538-5150; h https://orcid.org/0000-0002-7746-3826; i https://orcid.org/0000-0001-8826-5182; j https://orcid.org/0000-0001-8356-0764; k https://orcid.org/0000-0002-9398-5202

**Keywords:** cervical cancer, risk factor awareness, symptom awareness, lay beliefs, South Africa, Zimbabwe

## Abstract

Incidence and mortality rates of cervical cancer remain high in Southern Africa (SA). We explored awareness of cervical cancer symptoms and risk factors, as well as risk lay beliefs among women recently diagnosed with cervical cancer from SA and Zimbabwe. Patients were asked to complete a locally validated questionnaire with unprompted, open-ended questions to assess awareness of cervical cancer symptoms and risk factors. Among 501 women (SA 285, Zimbabwe 216), 46% (229) were able to recall one or more symptoms (SA 24%, Zimbabwe 76%) and 19% (93) were able to recall one or more risk factors of cervical cancer (SA 27%, Zimbabwe 73%). In SA, factors associated with increased symptom awareness included higher education level (completion of secondary education compared to not completing secondary education; adjusted odds ratios (aOR) 2.74, 95% confidence interval (CI) 1.17–6.43) as well as living in urban and peri-urban areas compared to living in rural areas (Urban: aOR 2.98, 95% CI 1.35–6.80; Peri-urban: aOR 3.28, 95% CI 1.13–9.35). Having a self-reported history of a chronic condition was associated with lower risk factor awareness compared to not having a self-reported chronic condition (aOR 0.07, 95% CI 0.00–0.42). In Zimbabwe, those who self-reported living with HIV were more likely to know one or more risk factors compared to those without HIV (aOR 2.69, 95% CI 1.31–5.67). Overall, 90 (18%) women mentioned at least one lay belief about risk factors for cervical cancer, with the most reported being inserting herbs, creams or objects into the vagina (9%, *n* = 43). The low levels of cervical cancer awareness in two Southern African countries highlight the urgent need to improve cervical cancer awareness, as low levels of awareness can impact timely cancer diagnosis and limit the uptake of cervical cancer prevention programs.

## Introduction

In 2022, there were an estimated 660,000 incident cases of cervical cancer and 350,000 related deaths globally, resulting in cervical cancer being the fourth most diagnosed cancer and the fourth leading cause of cancer death in women [1]. Low- and middle-income countries (LMICs) account for greater than 80% of all incident cases, and 90% of deaths, highlighting both the uneven global distribution of cervical cancer, and that cervical cancer is a disease of poverty and inequality [2–4]. Despite a decrease in the global age-standardised incidence rate (ASIR) and age-standardised mortality rate (ASMR) between 1990 and 2019, Southern sub-Saharan Africa (SSA) was the only region globally in which both the ASIR and ASMR increased over the same two-decade period (1990–2019) [5]. The increasing trend of cervical cancer ASIR and ASMR in Southern Africa (SA) over time, coupled with the fact that this region had one of the highest ASIR (34.9 per 100,000 women years) and one of the highest ASMRs (20.4 per 100,000 women years) globally in 2022, indicates the significant burden and impact that cervical cancer is having, and will continue to have, in this region [1, 3, 6]. In 2022, the ASIR for cervical cancer in SA (33.2 per 100,000 women) was more than double that of the global ASIR (14.1 per 100,000), while Zimbabwe had an ASIR (68.2 per 100,000 women) that was almost five times that of the global ASIR [7].

Cervical cancer is a preventable disease with highly effective primary and secondary prevention strategies, such as the Human papillomavirus (HPV) vaccine and cervical cancer screening [8, 9]. However, these prevention strategies have not been implemented equitably worldwide [9]. In 2019, an estimated 80% of countries in Africa did not have a national cervical cancer screening program, while in 2023, only 54% of countries in Africa had the HPV vaccination included in their national immunisation program [10, 11]. Furthermore, many of the African countries with cervical cancer prevention services have poor population coverage and public uptake [9, 12, 13]. Despite both SA and Zimbabwe having nationwide cervical cancer screening and HPV vaccination programs, the rates of cervical cancer remain extremely high in both countries [14–16]. Low cervical cancer screening coverage has been reported in SA and Zimbabwe, and a lack of awareness of cervical cancer screening is believed to play a major role in the low uptake of cervical cancer screening by the public [17, 18].

Several studies conducted among women and communities in SSA have found that the knowledge of cervical cancer symptoms and risk factors is generally poor [19–22]. Evidence suggests that awareness of cervical cancer symptoms can improve help-seeking behaviours, resulting in earlier presentation to health care facilities and, possibly, diagnosis at an earlier and more treatable stage of disease [22, 23]. With an estimated 65%–85% of cervical cancer patients in SSA being diagnosed at an advanced stage of disease, the need for improved awareness around symptoms of cervical cancer is crucial [24]. Improving awareness of certain risk factors, such as HPV infection and the importance of cervical cancer screening, is also essential [25]. In a study across SA and Uganda, less than 1% of participants mentioned either HPV infection or a lack of cervical cancer screening as risk factors for cervical cancer, with similar findings reported in a study in Libya [23, 26].

Many of the studies conducted in SSA assessing cervical cancer awareness have been conducted at a community or population level. Comparatively, little is known about the level of cervical cancer awareness among women diagnosed with cervical cancer in SSA. The aim of this study was to determine cervical cancer symptom and risk factor awareness and describe risk lay beliefs about cervical cancer among women recently diagnosed with cervical cancer in SA and Zimbabwe.

## Methods

### Study design

This cross-sectional survey of women recently diagnosed with cervical cancer in SA and Zimbabwe is part of a larger study, titled NIHR Global Research Group on Advancing Early Diagnosis of Cancer in Southern Africa: African Women Awareness of CANcer (AWACAN)-ED (https://awacan.online/), which aimed to evaluate the time intervals from breast, cervical and colorectal cancer symptom awareness to referral and diagnosis and the factors influencing these intervals [27].

### Study setting

The study was conducted in SA and Zimbabwe, two Southern African countries.

## South Africa

SA, an upper middle-income country with a population of approximately 60.5 million, has a 3-tiered referral-based public health care system [28, 29]. Participants from SA were selected from two of the nine provinces, namely the Western Cape, one of the wealthier provinces and the Eastern Cape, one of the poorer provinces [23]. Participants were from one tertiary-level hospital in each province. In the Western Cape, participants were selected from one of the two tertiary-level public adult hospitals in the province. The selected Western Cape tertiary hospital is in an urban centre and has a drainage population of over 2 million people [30]. In the Eastern Cape, participants were selected from one of the four tertiary hospitals in the province. The selected Eastern Cape hospital is in a rural area and serves a catchment area of approximately 3 million people [31].

## Zimbabwe

Zimbabwe is an LMIC, with a population of 15.1 million, 61% of whom live in rural areas [32, 33]. Zimbabwe also has a 3-tiered referral-based public health care system. Participants from Zimbabwe were selected from 2 of the country’s 10 provinces, namely Harare Province and Bulawayo Province. Harare Province is the most densely populated province in Zimbabwe, with a total population of 2.4 million people. Bulawayo Province is the least populated province in the country, with a total population of 665,940 people [34]. In both Harare Province and Bulawayo Province, there are no secondary hospitals, and all patients with suspected cancer symptoms are referred to tertiary-level facilities. Participants were from all tertiary-level facilities in each province.

### Study population

Between September 2022 and November 2023, women recently diagnosed with cervical cancer at these tertiary-level health care facilities, were invited to participate in the study. ‘Recently diagnosed’ was defined as women who were diagnosed with cervical cancer in the preceding month and/or were within 4 weeks of receiving a treatment plan at a tertiary level facility. For inclusion, women had to be 18 years or older and meet the above definition of a recent cervical cancer diagnosis. Individuals with a previous history of any cancer and those unwilling or unable to provide consent were excluded from the study. At each site, a clinical team identified eligible patients, referring them to the field research team, who explained the study aim, and obtained consent from individuals willing to participate.

## Data collection measures

Data were collected by trained fieldworkers using hand-held tablets customised with a structured, validated questionnaire [35]. The questionnaire collected information on socio-demographic and recall of cervical cancer symptoms, risk factors and lay beliefs.

The following socio-demographic information was collected: age, relationship status, highest level of education, country, province, self-reported living context, employment status and information on household spending. The living context was based on where participants reported they lived. The peri-urban living context setting was defined as areas outside of urban zones, characterised by farming and industrial land. In each country, socioeconomic status was measured using household expenditure, calculated from the data collected on frequently purchased household items (1-month recall period) and infrequently purchased household items (12-month recall period). Major expenditure categories include health care, medical aid or insurance, food and groceries, rentals, utility, transportation, clothing, education and childcare. An annualised total household expenditure was calculated by multiplying all the reported monthly spending (frequent items) by 12 and adding this to the reported annual expenditure for infrequently purchased items. By dividing the total annualised household expenditure by the total household size, a per capita expenditure was calculated, which represented the welfare level of each member in the household. Individuals were categorised into socioeconomic quintiles using per capita household expenditure. This was done separately for each country. Quintile 1 represents the most deprived household or individual, while quintile 5 represented the richest quintile.

Information on participant’s self-reported medical history was also obtained, including previously diagnosed hypertension, diabetes, HIV/AIDs and tuberculosis, as well as details of cervical cancer screening and knowing anyone (family or friend) with cancer.

### Measures of cervical cancer awareness

Cervical cancer awareness was assessed using two open/unprompted questions [35]. For symptom awareness, participants were asked, ‘Please would you name as many symptoms or signs of cervical cancer/cancer of the mouth of the womb as you can think of?’. For risk factor awareness, participants were asked, ‘Please could you name as many things as you can think of that could increase any person’s chances of getting cervical cancer?’.

Fieldworkers transcribed the exact participant responses, and these responses were then compared to a list of evidence-based cervical cancer symptoms and risk factors taken from the AWACAN questionnaire (Table S1) [35]. Participants scored one point for each correct symptom and each correct risk factor identified. Points were aggregated to give each participant two separate scores, one for symptom awareness and one for risk factor awareness. For analysis, drawing on the approach used in a study conducted in a similar setting, these scores were dichotomised into correctly identified zero versus at least one symptom and risk factor of cervical cancer [23].

Risk lay beliefs were identified as responses that did not fit into any of the evidence-based cervical cancer risk factors from the reference list. Similar to symptom and risk factor awareness scores, participants scored one point for each risk factor lay belief identified and these scores were dichotomised into zero versus at least one risk lay belief.

### Statistical analysis

Data were analysed using R Studio Version 2023.06.2+561. Descriptive statistics were used to characterise socio-demographic information. Continuous variables, such as age, were expressed as median (with interquartile range) and categorical data were expressed as frequencies and percentages. Symptom and risk factor awareness was stratified by country. Due to the differences in socio-demographic factors between SA and Zimbabwe, separate bivariate and multivariable logistic regression analysis was performed for risk factor awareness and symptom awareness for each country. Model results were reported as adjusted odds ratios (aOR) with 95% confidence intervals (CI). The initial multivariable models were built using the *a priori method* of variable selection, in which variables were selected based on the literature [22, 23, 36–39]. These were age, relationship status, level of education, employment status, expenditure index, self-reported living context, known family or friend with cancer, self-reported HIV status and previous cervical cancer screening. Any additional variables that were statistically associated (*p*-value <0.05) with cervical cancer symptom or risk factor awareness in the bivariate analysis were included in the country-specific multivariable analysis.

### Ethical considerations

Ethics approval was obtained from the University of Cape Town, Faculty of Health Sciences Human Research Ethics Committee (HREC 921/2023). The parent study, titled NIHR Global Research Group on Advancing Early Diagnosis of Cancer in Southern Africa: AWACAN-ED, received ethical clearance from all relevant committees in both SA and Zimbabwe prior to commencement of the study. In SA, ethics clearance was obtained from the Eastern Cape Department of Health (EC_202111_007) and the University of Cape Town Health Research Ethics Committee (HREC 664/2021). In Zimbabwe, ethics clearance was obtained from the Medical Research Council of Zimbabwe (MRCZ/A/2831) and the Joint Research Ethics Committee for Parirenyatwa Group of Hospitals and the University of Zimbabwe, Faculty of Medicine and Health Sciences & Parirenyatwa Group of Hospitals (JREC/363/21). Informed consent was obtained from all participants who met the inclusion criteria and were willing to participate in the parent study.

## Results

### Participant profile

Overall, 501 women with cervical cancer participated in this study, 285 (57%) from SA and 216 (43%) from Zimbabwe (Table 1). The median age was similar in both countries (49.0 (IQR 41.2–58.7) in SA and 51.0 (IQR 44.8–61.7) in Zimbabwe, respectively).

A similar proportion of women were unemployed in both countries (76% in SA and 69% in Zimbabwe). SA had a far higher proportion of single women (37% versus 7%) and a lower proportion of separated, divorced or widowed women (30% versus 49%) compared to Zimbabwe. The proportion of women in Zimbabwe who completed secondary school was almost double that of SA (32% versus 17%, respectively). Zimbabwe had a higher proportion of women living in urban areas (51% versus 37%) compared to SA; however, the proportion of women who fell into each expenditure index quintile was similar in both countries. The proportion of women who self-reported previously attending screening for cervical cancer was higher in SA compared to Zimbabwe (93% versus 79%). Of the 264 women in SA who reported having been previously screened for cervical cancer, 94% (*n* = 248) were screened within the last year and 70% (*n* = 184) received a cytology screening test. In Zimbabwe, of the 170 women who reported having been previously screened for cervical cancer, 75% (*n* = 127) were screened in the last year and almost all received the visual inspection method of screening (97%).

### Symptom awareness

Overall, 229 (46%) women were able to recall at least one symptom of cervical cancer (Table 2). The most recalled symptoms in the overall sample were vaginal discharge or smelly vaginal discharge (26%, *n* = 129), vaginal or lower abdominal/pelvic pain (20%, *n* = 99) and lower back pain (8%, *n* = 41) (Table S2). In SA, the most recalled symptom was vaginal or lower abdominal/pelvic pain (10%, *n* = 29), followed by vaginal discharge or smelly vaginal discharge (9%, *n* = 25). In Zimbabwe, the most recalled symptom was vaginal discharge or smelly vaginal discharge (48%, *n* = 104), followed by vaginal or lower abdominal/pelvic pain (32%, *n* = 70) (Table S2). Overall, few women mentioned the specific cervical cancer symptoms related to vaginal bleeding (intermenstrual bleeding 4%, longer or heavier menstrual periods 1%, post-menopausal bleeding 7%, vaginal bleeding during or after sex 3%).

Table 2 describes cervical cancer symptom awareness stratified by country. 81% (*n* = 174) of participants in Zimbabwe and 19% (*n* = 55) of those in SA recalled at least one cervical cancer symptom. In SA, 35% of women who were able to recall at least one symptom had completed secondary school, compared to only 13% of those who were not able to recall any symptoms (*p* = < 0.001) (Table 2).

Among South African women, age, level of education, expenditure index, self-reported living context and knowing a family member or friend with cancer were all associated with symptom awareness in bivariate analysis (Table 3). In a multivariable logistic regression including these covariates, level of education and self-reported living context were the only factors that remained significantly associated with symptom awareness. Women who completed secondary education or reported living in an urban or peri-urban area were statistically significantly more likely to know at least one cervical cancer symptom compared to those with less than secondary school education (aOR 2.74 95% CI 1.17–6.43) or those who reported living in a rural setting (Urban: aOR 2.98 95% CI 1.35–6.80; Peri-urban: aOR 3.28 95% CI 1.13–9.35). Among women in Zimbabwe, the expenditure index was the only factor that was significantly associated with symptom awareness. Zimbabwean women who fell into the 2nd poorest expenditure index quintile were statistically significantly less likely to know at least one cervical cancer symptom compared to those who fell into the 1st quintile (aOR 0.20 95% CI 0.05–0.61) (Table 3 and S3).

### Risk factor awareness

Overall, 93 (19%) women were able to name at least one correct risk factor for cervical cancer (Table 4). The most recalled risk factors in the overall sample were having many sexual partners or partner having many sexual partners (8%, *n* = 40), having unprotected sexual intercourse (3%, *n* = 17) and HIV/AIDs (3%, *n* = 17) (Table S2). In SA, the most recalled risk factors were smoking cigarettes (5%, *n* = 13), not going for regular screening (2%, *n* = 5) and unprotected sexual intercourse (2%, *n* = 5). In Zimbabwe, the most recalled risk factors were having many sexual partners or partner having many sexual partners (17%, *n* = 36), HIV/AIDs (7%, *n* = 16) and having unprotected sex (6%, *n* = 12) (Table S2).

Table 4 describes cervical cancer risk factor awareness stratified by country. 31% (*n* = 68) of participants in Zimbabwe and 9% (*n* = 25) in SA recalled at least one cervical cancer risk factor. In both SA and Zimbabwe, the majority of women with no cervical cancer risk factor awareness were unemployed (77% in SA and 76% in Zimbabwe) (Table 4). In SA, 44% of women who recalled at least one risk factor had completed secondary school education, compared to only 15% of those who were not able to recall any risk factors (*p* = <0.001) (Table 4). This finding was similar in Zimbabwe, with 43% of women who were able to recall at least one risk factor having completed secondary school education, compared to only 28% of women who were not able to recall any risk factors (*p* = 0.043). The proportion of women who had previously been screened for cervical cancer were similar in both groups (Table 4).

Among women in SA, level of education, self-reported living context, self-reported history of any chronic conditions and expenditure index were all associated with cervical cancer risk factor awareness in bivariate analysis (Table 5). However, in a multivariable logistic regression including these variables, having any self-reported chronic condition was the only factor that remained significantly associated with risk factor awareness (Table 5). Those who reported having a chronic condition were less likely to know at least one cervical cancer risk factor compared to those who reported not having any chronic conditions (aOR 0.07 95% CI 0.00–0.42). Among women in Zimbabwe, level of education, employment status, self-reported HIV status and self-reported previously screening for cervical cancer were associated with cervical cancer risk factor awareness in bivariate analysis (Table 5). Self-reported HIV status was the only factor that remained significantly associated in the multivariable model. Women who self-reported living with HIV were more likely to know at least one cervical cancer risk factor compared to those who reported not living with HIV (aOR 2.69 95% CI 1.31–5.67) (Table 5 and S4).

### Lay beliefs

**Figure 1.**
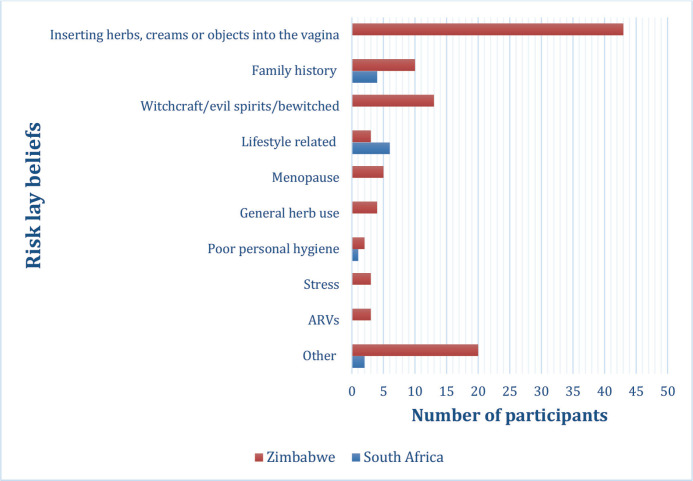
Bar graph showing cervical cancer risk lay beliefs, stratified by country.

Overall, 90 (18%) women mentioned at least one lay belief about risk factors for cervical cancer (Table S2), with most reported by women from Zimbabwe (Figure 1). The most reported risk lay belief, inserting herbs, creams or objects into the vagina (9%, *n* = 43), was only reported by women from Zimbabwe (Table S4).

## Discussion

To the best of our knowledge, this is the first study conducted in Southern Africa that determined the levels of awareness of cervical cancer symptoms and risk factors among women recently diagnosed with cervical cancer, using a locally validated questionnaire. We found low levels of cervical cancer awareness, particularly among women in SA. For both symptom and risk factor awareness, a far higher proportion of women from Zimbabwe were able to recall at least one symptom or risk factor of cervical cancer compared to women from SA. Among South African women, the level of education and self-reported living context were associated with symptom awareness, while having a chronic condition was associated with less risk factor awareness. Among Zimbabwean women, the expenditure index was associated with symptom awareness, while self-reported HIV was associated with risk factor awareness.

The finding of low cervical cancer symptom awareness in our study was consistent with findings in other studies conducted across Africa [20, 21, 23, 26]. However, a major difference between our study and many other studies conducted across Africa was the study population. Our study was conducted among women diagnosed with cervical cancer, while many other studies were conducted at a community level. One would expect women diagnosed with cervical cancer to have more cervical cancer symptom awareness. In a study conducted among South African and Ugandan women, only 58% of women were able to recall one correct symptom of cervical cancer, while a study in Libya found that 63% of women were not able to recall any signs or symptoms of cervical cancer [23, 26]. Both studies [23, 26] were population based, and used a validated questionnaire, assessing symptom awareness with open-ended questions. Interestingly, other studies conducted in Africa have found high levels of symptom awareness for cervical cancer. For example, a population-based study set in Northern Uganda, conducted among men and women without cervical cancer, found that most participants recognised cervical cancer symptoms [22]. However, the latter study tested recognition of symptoms by providing cues or limited response options, and is therefore prone to participants guessing, potentially resulting in higher recognition scores [23].

In our study, overall symptom recall was poor, with the most recalled symptoms being vaginal discharge or smelly vaginal discharge and vaginal or lower abdominal/pelvic pain. These two symptoms were commonly recalled or recognised in other studies conducted across Africa [21–24]. Interestingly, in our study, far fewer women were able to recall cervical cancer symptoms related to vaginal bleeding compared to other studies in Africa. In the above-mentioned studies conducted in Libya and across SA and Uganda, the most recalled symptom in both studies was vaginal bleeding between periods. In Libya, 22% of women and, in SA and Uganda, 28% of women were able to recall this symptom [23, 26]. In our study, intermenstrual bleeding was one of the least recalled symptoms, with only 4% of women recalling this symptom. For several studies conducted in Africa, which tested symptom recognition, intermenstrual bleeding and post-menopausal bleeding were among the most recognised cervical cancer symptoms [23–26]. Considering that, in our study, women were diagnosed with cervical cancer and were likely symptomatic, it is concerning that so few women were able to recall common symptoms of cervical cancer. This highlights the urgent need to address and improve the awareness around cervical cancer symptoms in Southern Africa. Symptom awareness is a crucial first step in the pathway to cancer care for every patient. Thus, improving cervical cancer symptom awareness can increase the likelihood of patients seeking care, leading to a timelier diagnosis with improved outcomes [23, 40].

Similar to our SA findings, the association between higher levels of education and greater cervical cancer knowledge was also reported in studies conducted in Ethiopia and Cameroon [36]. This finding emphasises that education and health literacy essential in improving the level of cervical cancer symptom and risk factor awareness among women. However, the 2022 global education monitoring report highlighted the gender inequality in the access to and completion of education, with one in four women from SSA unable to read and write. There is therefore an urgent need for a multi-sectoral approach, which includes improving access to education for women, to address the low levels of cervical cancer awareness in SSA countries [20, 21, 23, 40].

Our study highlighted that women from SA, who reported living in rural settings, had lower awareness of cervical cancer symptoms. SA is a country with extreme inequity, evidenced by the Gini coefficient of 0.6 and a large population of individuals living in rural areas [23, 41, 42]. Women living in rural areas are likely to have less access to evidence-based cervical cancer information as well as screening facilities compared to women in urban settings [12, 23]. It is therefore necessary to implement cervical cancer education and awareness interventions in rural communities as well as in primary health care facilities, which are the entry point into the South African healthcare system.

There is previous evidence of low levels of knowledge of cervical cancer risk factors in most SSA countries, including Kenya, Zimbabwe, Cameroon and Nigeria [22]. A study in Nigeria, which used a structured, unvalidated questionnaire among women attending antenatal and gynaecological outpatient clinics, found that only 16% of women had good knowledge of cervical cancer risk factors. However, in this study, good knowledge of risk factors was defined as being able to correctly identify more than 6 out of the 12 risk factors of cervical cancer provided [21]. A study among South African female university students found that, of the 43% of participants who had heard of cervical cancer, 16% did not know any risk factors for cervical cancer, while a separate study in Zimbabwe found that only 1% of female university students were knowledgeable about cervical cancer risk factors [43, 44].

HPV vaccination and cervical cancer screening are key interventions, and in countries with effective, long-standing cervical cancer prevention programs, the rates of cervical cancer are very low [5, 45–48]. In LMICs, a lack of knowledge around cervical cancer, and in particular, the association between HPV and cervical cancer, is a major barrier to the uptake of cervical cancer prevention interventions [49, 50]. Alarmingly,

in our study, very few women mentioned HPV infection or not going for screening as a cervical cancer risk factor (0.8% and 1% respectively). Similarly, low levels of awareness for these two risk factors were found in a community-based study among women conducted in SA and Uganda, which also used a validated questionnaire and tested unprompted (recall) risk factor awareness [23]. Studies conducted in Ethiopia and Kenya have also found a lack of awareness of screening as a risk factor for cervical cancer [23, 51, 52].

Both SA, an UMIC and Zimbabwe, an LMIC, have a referral-based public healthcare system, a nationwide cervical cancer screening program and an HPV vaccination program; however, the rates of cervical cancer remain high in both these countries [14–16]. Nationwide HPV vaccination programs have recently been introduced into both countries (2014 in SA and 2018 in Zimbabwe) [53, 54]. However, SA is already experiencing a decline in the rate of young girls getting vaccinated [53]. Low levels of knowledge and awareness around the role of HPV infection can potentially contribute to decreasing participation in intervention programs, such as HPV vaccination [25]. Therefore, introducing and implementing intervention programs alone is not enough and governments also need to address and improve the low levels of awareness and knowledge around cervical cancer, to ensure high uptake and utilisation of prevention programs [25].

A crucial factor contributing to the high burden of cervical cancer in Southern Africa is the high prevalence of HIV in this region [4, 13]. Both SA and Zimbabwe have high HIV burdens, with SA accounting for approximately 20% of all people living with HIV globally [55, 56]. In SA, 53% of all cervical cancer cases are attributable to HIV [57]. One would expect symptom and risk factor awareness to be higher among women living with HIV considering the frequent visits to health care facilities, providing opportunities for health education. However, our study found that this was not the case, as self-reported living with HIV was not associated with cervical cancer symptom awareness in both countries and was only significantly associated with risk factor awareness among Zimbabwean women. Considering that women living with HIV are believed to be at a 6-times higher risk of developing cervical cancer compared to women without HIV, it is essential to incorporate health education and promotion of cervical cancer awareness at every health encounter, particularly for women living with HIV [4, 13].

The most reported lay belief in our study, inserting herbs, creams or objects into the vagina as a cause of cervical cancer (9%, *n* = 43), has also been reported in other studies in Africa [23]. It is important to understand and identify lay beliefs, as they can potentially negatively impact other disease prevention and public health interventions. For example, a few women (<1%) in our study mentioned antiretroviral (ARVs) drugs as a cause of cervical cancer. This lay belief can increase non-adherence to ARVs, even though ARVs have been proven to be effective in managing HIV. Additionally, women who have a lay belief, such as inserting herbs, creams or objects into the vagina is a cervical cancer risk factor, might believe that if they do not engage in this behaviour, they might not be at risk of developing cervical cancer. Understanding lay beliefs around cervical cancer is necessary, especially when designing and implementing healthcare interventions. It is essential that, in addition to improving awareness and knowledge around cervical cancer, health promotion programs and interventions also address and correct lay beliefs [23]. Framing health interventions around the beliefs of the individuals and communities who are going to utilise the intervention, increases the likelihood that the intervention will be accepted by the community, thereby increasing the uptake and effectiveness of the intervention [58].

### Limitations

A major strength of our study was that cervical cancer symptom and risk factor awareness was measured using a questionnaire that was locally validated. Additionally, our study tested unprompted recall of symptoms and risk factors, which has been shown to be a more accurate representation of knowledge and awareness compared to recognition of symptoms and risk factors [23, 26].

A limitation of our study was that, due to the low levels of awareness in our study, both symptom and risk factor awareness was defined as being able to mention at least one correct symptom or risk factor for cervical cancer. This meant that even women who were only able to recall one symptom or risk factor were still classified as having risk factor or symptom awareness. As a result, we were only able to determine factors associated with knowing at least one symptom or risk factor. Another potential limitation in our study was enrolling women who were recently diagnosed with cervical cancer. A diagnosis of cancer can cause significant psychological distress, and although attempts were made to ensure that no participant was interviewed while showing signs of distress, the psychological effect of a cancer diagnosis could have resulted in limited participant engagement and responses during the interview. Additionally, participants could have underreported awareness to avoid appearing as if they ignored symptoms or delayed help-seeking behaviour. Lastly, by only enrolling women who were diagnosed with cervical cancer, the findings of this study may not be generalisable to the general population.

## Conclusion

This study highlights the low levels of cervical cancer symptom and risk factor awareness in two Southern African countries among women recently diagnosed with cervical cancer and emphasises the dire need to implement and initiate health interventions in SA and Zimbabwe addressing the low levels of cervical cancer awareness. Improved symptom awareness can lead to earlier help-seeking behaviour resulting in earlier presentation to health care facilities, and potentially an earlier stage of disease diagnosis. Meanwhile, improved risk factor awareness, in particular HPV infection and screening, can prevent the onset of cervical cancer in women. Improving both symptom and risk factor awareness is essential to reducing the high burden of this disease in Southern Africa.

## Conflicts of interest

The authors declare no conflicts of interest.

## Figures and Tables

**Table 1.** Overall participant profile, stratified by country.

Characteristics	Overall, *N* = 501 *n* (%)	SA, *N* = 285 *n* (%)	Zimbabwe, *N* = 216 *n* (%)
Age, (years)			
18–34	30 (6.0)	22 (7.7)	8 (3.7)
35–44	130 (26.0)	80 (28.2)	50 (23.1)
45–54	161 (32.2)	87 (30.6)	74 (34.3)
>55	179 (35.8)	95 (33.5)	84 (38.9)
Median (IQR)	50.2 (42.9–59.8)	49.0 (41.2–58.7)	51.0 (44.8–61.7)
Province residing in			
Western Cape	119 (23.8)	119 (41.8)	
Eastern Cape	166 (33.1)	166 (58.2)	
Harare and referral provinces	142 (28.3)		142 (65.7)
Bulawayo and referral provinces	74 (14.8)		74 (34.3)
Relationship status			
Married/living with a partner	191 (38.2)	96 (33.7)	95 (44.2)
Single	119 (23.8)	105 (36.8)	14 (6.5)
Separated/divorced/widowed	190 (38.0)	84 (29.5)	106 (49.3)
Education			
Less than secondary school	382 (76.2)	236 (82.8)	146 (67.6)
Secondary school completed	119 (23.8)	49 (17.2)	70 (32.4)
Employment			
Employed	136 (27.1)	69 (24.2)	67 (31.0)
Unemployed	365 (72.9)	216 (75.8)	149 (69.0)
Country specific expenditure index			
First quintile (poorest)		75 (26.5)	48 (22.2)
Second quintile		76 (26.9)	51 (23.6)
Third quintile (middle)		56 (19.8)	41 (19.0)
Fourth quintile		43 (15.2)	42 (19.4)
Fifth quintile (richest)		33 (11.7)	34 (15.7)
Self-report living context			
Rural	243 (48.5)	150 (52.6)	93 (43.1)
Urban	218 (43.5)	107 (37.5)	111 (51.4)
Peri-urban	40 (8.0)	28 (9.8)	12 (5.6)
Known family member or friend with cancer			
No	333 (66.5)	188 (66.0)	145 (67.1)
Yes	168 (33.5)	97 (34.0)	71 (32.9)
Self-reported known HIV			
No	229 (45.7)	134 (47.0)	95 (44.0)
Yes	272 (54.3)	151 (53.0)	121 (56.0)
Self-reported history of any chronic disease *			
No	114 (22.8)	67 (23.5)	47 (21.8)
Yes	387 (77.2)	218 (76.5)	169 (78.2)
Self-reported previous cervical cancer screening			
No	66 (13.2)	21 (7.4)	45 (20.9)
Yes	434 (86.8)	264 (92.6)	170 (79.1)
Last screened for cervical cancer			
<1 year ago	375 (86.4)	248 (93.9)	127 (74.7)
1–5 years ago	47 (10.8)	15 (5.7)	32 (18.8)
> 6 years ago	12 (2.8)	1 (0.4)	11 (6.5)
Type of last cervical cancer screening test			
Cytology	185 (42.6)	184 (69.7)	1 (0.6)
Visual inspection	179 (41.2)	14 (5.3)	165 (97.1)
HPV test	38 (8.8)	37 (14.0)	1 (0.6)
Combination	8 (1.8)	5 (1.9)	3 (1.8)
Not sure of test	24 (5.5)	24 (9.1)	0 (0.0)

IQR – Interquartile range

* Chronic disease include hypertension, diabetes, HIV, cardiac disease

**Table 2.** Cervical cancer symptom awareness overall and stratified by country.

	Overall		SA		Zimbabwe	
Characteristics	Symptom awareness score: Zero *N* = 272 *n* (%)	Symptom awareness score: At least one *N* = 229 *n* (%)	Symptom awareness score: Zero *N* = 230 *n* (%)	Symptom awareness score: At least one *N* = 55 *n* (%)	Symptom awareness score: Zero *N* = 42 *n* (%)	Symptom awareness score: At least one *N* = 174 *n* (%)
Age (years)						
18–34	18 (6.6)	12 (5.2)	16 (7.0)	6 (10.9)	2 (4.8)	6 (3.4)
35–44	68 (25.1)	62 (27.1)	58 (25.3)	22 (40.0)	10 (23.8)	40 (23.0)
45–54	85 (31.7)	76 (33.2)	69 (30.1)	18 (32.7)	16 (38.1)	58 (33.3)
>55	100 (36.9)	79 (34.5)	86 (37.6)	9 (16.4)	14 (33.3)	70 (40.2)
Median (IQR)	50.2 (43.2–59.5)	50.1 (42.6–60.3)	50.3 (43.0; 59.6)	44.3 (38.2; 51.6)	49.4 (44.3; 59.1)	52.0 (44.8; 61.8)
Relationship						
Married/living with partner	92 (33.8)	99 (43.4)	73 (31.7)	23 (41.8)	19 (45.2)	76 (43.9)
Single	88 (32.4)	31 (13.6)	84 (36.5)	21 (38.2)	4 (9.5)	10 (5.8)
Separated/divorced/widowed	92 (33.8)	98 (43.0)	73 (31.7)	11 (20.0)	19 (45.2)	87 (50.3)
Education						
Less than secondary school	229 (84.2)	153 (66.8)	200 (87.0)	36 (65.5)	29 (69.0)	117 (67.2)
Secondary school completed	43 (15.8)	76 (33.2)	30 (13.0)	19 (34.5)	13 (31.0)	57 (32.8)
Employment						
Unemployed	205 (75.4)	160 (69.9)	176 (76.5)	40 (72.7)	29 (69.0)	120 (69.0)
Employed	67 (24.6)	69 (30.1)	54 (23.5)	15 (27.3)	13 (31.0)	54 (31.0)
Expenditure index						
First quintile (poorest)			66 (28.8)	9 (16.7)	4 (9.5)	44 (25.3)
Second quintile			63 (27.5)	13 (24.1)	16 (38.1)	35 (20.1)
Third quintile (middle)			41 (17.9)	15 (27.8)	7 (16.7)	34 (19.5)
Fourth quintile			36 (15.7)	7 (13.0)	10 (23.8)	32 (18.4)
Fifth quintile (richest)			23 (10.0)	10 (18.5)	5 (11.9)	29 (16.7)
Self-reported living context						
Rural	155 (57.0)	88 (38.4)	136 (59.1)	14 (25.5)	19 (45.2)	74 (42.5)
Urban	98 (36.0)	120 (52.4)	76 (33.0)	31 (56.4)	22 (52.4)	89 (51.1)
Peri-urban	19 (7.0)	21 (9.2)	18 (7.8)	10 (18.2)	1 (2.4)	11 (6.3)
Known family or friend with cancer						
No	186 (68.4)	147 (64.2)	160 (69.6)	28 (50.9)	26 (61.9)	119 (68.4)
Yes	86 (31.6)	82 (35.8)	70 (30.4)	27 (49.1)	16 (38.1)	55 (31.6)
Self-reported known with HIV						
No	124 (45.6)	105 (45.9)	106 (46.1)	28 (50.9)	18 (42.9)	77 (44.3)
Yes	148 (54.4)	124 (54.1)	124 (53.9)	27 (49.1)	24 (57.1)	97 (55.7)
Self-reported history of any chronic disease*						
No	62 (22.8)	52 (22.7)	51 (22.2)	16 (29.1)	11 (26.2)	36 (20.7)
Yes	210 (77.2)	177 (77.3)	179 (77.8)	39 (70.9)	31 (73.8)	138 (79.3)
Self-reported previous cervical cancer screening						
No	25 (9.2)	41 (17.9)	17 (7.4)	4 (7.3)	8 (19.5)	37 (21.3)
Yes	246 (90.8)	188 (82.1)	213 (92.6)	51 (92.7)	33 (80.5)	137 (78.7)
Risk factor awareness ^#^						
No	255 (93.8)	153 (66.8)	220 (95.7)	40 (72.7)	35 (83.3)	113 (64.9)
Yes	17 (6.2)	76 (33.2)	10 (4.3)	15 (27.3)	7 (16.7)	61 (35.1)

IQR – Interquartile range

* Chronic diseases include hypertension, diabetes, HIV, cardiac disease

# Recalling at least one correct risk factor for cervical cancer

**Table 3.** Factors associated with cervical cancer symptom awareness in each country.

	SA		Zimbabwe	
Characteristics	Crude OR (95% CI)	aOR (95% CI)	Crude OR (95% CI)	aOR (95% CI)
Age				
18–34	Ref	Ref	Ref	Ref
35–44	1.01 (0.36; 3.12)	1.03 (0.32; 3.67)	1.33 (0.18; 6.87)	1.20 (0.15; 6.90)
45–54	0.70 (0.25; 2.16)	0.75 (0.22; 2.72)	1.21 (0.17; 5.85)	0.87 (0.11; 4.66)
>55	**0.28 (0.09; 0.93)**	0.29 (0.07; 1.18)	1.67 (0.23; 8.14)	1.38 (0.17; 7.98)
Relationship				
Married/living with partner	Ref	Ref	Ref	Ref
Single	0.79 (0.40; 1.55)	0.65 (0.29; 1.44)	0.62 (0.19; 2.47)	0.89 (0.21; 4.62)
Separated/divorced/widowed	0.48 (0.21; 1.03)	0.84 (0.33; 2.10)	1.14 (0.56; 2.33)	1.02 (0.46; 2.26)
Education				
Less than secondary school	Ref	Ref	Ref	Ref
Secondary school completed	**3.52 (1.78; 6.90)**	**2.74 (1.17; 6.43)**	1.09 (0.53; 2.31)	1.07 (0.42; 2.78)
Employment				
Unemployed	Ref	Ref	Ref	Ref
Employed	1.22 (0.61; 2.34)	0.78 (0.34; 1.70)	1.00 (0.49; 2.14)	1.07 (0.45; 2.65)
Country specific expenditure index				
First quintile (poorest)	Ref	Ref	Ref	Ref
Second quintile	1.51 (0.61; 3.90)	1.27 (0.47; 3.52)	**0.20 (0.05; 0.60)**	**0.20 (0.05; 0.61)**
Third quintile (middle)	**2.68 (1.09; 6.92)**	1.74 (0.65; 4.85)	0.44 (0.11; 1.58)	0.43 (0.10; 1.64)
Fourth quintile	1.43 (0.47; 4.15)	0.73 (0.22; 2.38)	**0.29 (0.07; 0.95)**	0.30 (0.07; 1.17)
Fifth quintile (richest)	**3.19 (1.15; 9.01)**	1.03 (0.27; 3.82)	0.53 (0.12; 2.15)	0.53 (0.10; 2.55)
Self-reported living context				
Rural	Ref	Ref	Ref	Ref
Urban	**3.96 (2.02; 8.12)**	**2.98 (1.35; 6.80)**	1.04 (0.52; 2.06)	1.05 (0.44; 2.53)
Peri-urban	**5.40 (2.06; 13.98)**	**3.28 (1.13; 9.35)**	2.82 (0.50; 53.22)	2.32 (0.37; 45.32)
Known family member or friend with cancer				
No	Ref	Ref	Ref	Ref
Yes	**2.20 (1.21; 4.02)**	1.59 (0.79; 3.19)	0.75 (0.38; 1.54)	0.78 (0.37; 1.68)
Self-reported known HIV				
No	Ref	Ref	Ref	Ref
Yes	0.82 (0.46; 1.49)	0.85 (0.39; 1.84)	0.94 (0.47; 1.86)	1.22 (0.54; 2.74)
Self-reported previous cervical cancer screening				
No	Ref	Ref	Ref	Ref
Yes	1.02 (0.36; 3.65)	0.89 (0.26; 3.68)	0.90 (0.36; 2.03)	1.07 (0.39; 2.70)

OR – odds ratio

CI – confidence interval

Bold OR & CI – variable is statistically significant

**Table 4.** Cervical cancer risk factor awareness stratified by country.

	Overall		SA		Zimbabwe	
Characteristics	Risk factor awareness score: Zero *N* = 408 *n* (%)	Risk factor awareness score: At least one *N* = 93 *n* (%)	Risk factor awareness score: Zero *N* = 260 *n* (%)	Risk factor awareness score: At least one *N* = 25 *n* (%)	Risk factor awareness score: Zero *N* = 148 *n* (%)	Risk factor awareness score: At least one *N* = 68 *n* (%)
Age, (years)						
18–34	26 (6.4)	4 (4.3)	20 (7.7)	2 (8.0)	6 (4.1)	2 (2.9)
35–44	100 (24.6)	30 (32.3)	70 (27.0)	10 (40.0)	30 (20.3)	20 (29.4)
45–54	125 (30.7)	36 (38.7)	79 (30.5)	8 (32.0)	46 (31.1)	28 (41.2)
>55	156 (38.3)	23 (24.7)	90 (34.7)	5 (20.0)	66 (44.6)	18 (26.5)
Median (IQR)	51.0 (43.8; 61.0)	47.4 (40.4; 54.0)	49.8 (41.9; 58.9)	45.2 (39.5; 51.8)	53.4 (45.4; 63.0)	48.1 (41.6; 57.9)
Relationship status						
Married/living with a partner	144 (35.4)	47 (50.5)	84 (32.3)	12 (48.0)	60 (40.8)	35 (51.5)
Single	105 (25.8)	14 (15.1)	97 (37.3)	8 (32.0)	8 (5.4)	6 (8.8)
Separated/divorced/widowed	158 (38.8)	32 (34.4)	79 (30.4)	5 (20.0)	79 (53.7)	27 (39.7)
Education						
Less than secondary school	329 (80.6)	53 (57.0)	222 (85.4)	14 (56.0)	107 (72.3)	39 (57.4)
Secondary school completed	79 (19.4)	40 (43.0)	38 (14.6)	11 (44.0)	41 (27.7)	29 (42.6)
Employment						
Unemployed	313 (76.7)	52 (55.9)	200 (76.9)	16 (64.0)	113 (76.4)	36 (52.9)
Employed	95 (23.3)	41 (44.1)	60 (23.1)	9 (36.0)	35 (23.6)	32 (47.1)
Country specific expenditure index						
First quintile (poorest)			71 (27.5)	4 (16.0)	35 (23.6)	13 (19.1)
Second quintile			70 (27.1)	6 (24.0)	40 (27.0)	11 (16.2)
Third quintile (middle)			51 (19.8)	5 (20.0)	28 (18.9)	13 (19.1)
Fourth quintile			39 (15.1)	4 (16.0)	25 (16.9)	17 (25.0)
Fifth quintile (richest)			27 (10.5)	6 (24.0)	20 (13.5)	14 (20.6)
Self-reported living context						
Rural	213 (52.2)	30 (32.3)	144 (55.4)	6 (24.0)	69 (46.6)	24 (35.3)
Urban	165 (40.4)	53 (57.0)	94 (36.2)	13 (52.0)	71 (48.0)	40 (58.8)
Peri-urban	30 (7.4)	10 (10.8)	22 (8.5)	6 (24.0)	8 (5.4)	4 (5.9)
Known family member or friend with cancer						
No	278 (68.1)	55 (59.1)	173 (66.5)	15 (60.0)	105 (70.9)	40 (58.8)
Yes	130 (31.9)	38 (40.9)	87 (33.5)	10 (40.0)	43 (29.1)	28 (41.2)
Self-reported known HIV						
No	195 (47.8)	34 (36.6)	118 (45.4)	16 (64.0)	77 (52.0)	18 (26.5)
Yes	213 (52.2)	59 (63.4)	142 (54.6)	9 (36.0)	71 (48.0)	50 (73.5)
Self-reported history of any chronic disease*						
No	89 (21.8)	25 (26.9)	52 (20.0)	15 (60.0)	37 (25.0)	10 (14.7)
Yes	319 (78.2)	68 (73.1)	208 (80.0)	10 (40.0)	111 (75.0)	58 (85.3)
Self-reported previous cervical cancer screening						
No	56 (13.7)	10 (10.9)	19 (7.3)	2 (8.0)	37 (25.0)	8 (11.9)
Yes	352 (86.3)	82 (89.1)	241 (92.7)	23 (92.0)	111 (75.0)	59 (88.1)
Symptom awareness^$^						
No	255 (62.5)	17 (18.3)	220 (84.6)	10 (40.0)	35 (23.6)	7 (10.3)
Yes	153 (37.5)	76 (81.7)	40 (15.4)	15 (60.0)	113 (76.4)	61 (89.7)

IQR – Interquartile range

* Chronic diseases include hypertension, diabetes, HIV, cardiac disease

$ Recalling at least one correct cervical cancer symptom

**Table 5.** Factors associated with cervical cancer risk factor awareness in each country.

	SA		Zimbabwe	
Characteristic	Crude OR (95% CI)	aOR (95% CI)	Crude OR (95% CI)	aOR (95% CI)
Age, (years)				
18–34	Ref	Ref	Ref	Ref
35–44	1.43 (0.34; 9.79)	3.27 (0.58; 28.74)	2.00 (0.41; 14.59)	1.14 (0.21; 9.14)
45–54	1.01 (0.23; 7.06)	2.39 (0.39; 21.78)	1.83 (0.39; 13.05)	1.39 (0.26; 10.89)
>55	0.56 (0.11; 4.07)	1.86 (0.25; 18.70)	0.82 (0.17; 5.91)	1.05 (0.19; 8.39)
Relationship				
Married/living with partner	Ref	Ref	Ref	Ref
Single	0.58 (0.22; 1.46)	0.81 (0.26; 2.52)	1.29 (0.39; 4.00)	0.80 (0.20; 3.02)
Separated/divorced/widowed	0.44 (0.14; 1.25)	0.76 (0.20; 2.69)	0.59 (0.32; 1.07)	0.57 (0.28; 1.16)
Education				
Less than secondary school	Ref	Ref	Ref	Ref
Secondary school completed	**4.59 (1.91; 10.86)**	2.96 (0.90; 9.50)	**1.94 (1.06; 3.54)**	1.32 (0.59; 2.90)
Employment				
Unemployed	Ref	Ref	Ref	Ref
Employed	1.87 (0.76; 4.38)	1.16 (0.39; 3.24)	**2.87 (1.56; 5.30)**	1.83 (0.88; 3.81)
Country specific expenditure index				
First quintile (poorest)	Ref	Ref	Ref	Ref
Second quintile	1.52 (0.42; 6.17)	1.30 (0.31; 5.89)	0.74 (0.29; 1.86)	0.53 (0.18; 1.48)
Third quintile (middle)	1.74 (0.44; 7.33)	1.35 (0.29; 6.54)	1.25 (0.50; 3.15)	0.94 (0.33; 2.64)
Fourth quintile	1.82 (0.41; 8.09)	1.23 (0.23; 6.41)	1.83 (0.76; 4.51)	0.87 (0.29; 2.56)
Fifth quintile (richest)	**3.94 (1.05; 16.47)**	0.89 (0.14; 5.38)	1.88 (0.74; 4.85)	1.01 (0.31; 3.25)
Self-report living context				
Rural	Ref	Ref	Ref	Ref
Urban	**3.32 (1.26; 9.73)**	2.01 (0.61; 7.00)	1.62 (0.89; 2.99)	0.94 (0.42; 2.06)
Peri-urban	**6.55 (1.90; 22.74)**	4.12 (0.97; 17.21)	1.44 (0.36; 5.01)	0.83 (0.17; 3.51)
Known family member or friend with cancer				
No	Ref	Ref	Ref	Ref
Yes	1.33 (0.56; 3.04)	0.74 (0.25; 2.04)	1.71 (0.94; 3.11)	1.72 (0.88; 3.35)
Self-reported known HIV				
No	Ref	Ref	Ref	Ref
Yes	0.47 (0.19; 1.08)	3.20 (0.51; 63.04)	**3.01 (1.63; 5.76)**	**2.69 (1.31; 5.67)**
Self-reported previous cervical cancer screening				
No	Ref	Ref	Ref	Ref
Yes	0.91 (0.24; 5.91)	0.52 (0.11; 3.87)	**2.46 (1.12; 5.99)**	1.57 (0.65; 4.15)
Self-reported history of any chronic conditions				
No	Ref	Ref		
Yes	**0.16 (0.07; 0.38)**	**0.07 (0.00; 0.42)**		

OR – odds ratio

CI – confidence interval

Bold OR & CI – variable is statistically significant

## Supplementary Table

**Table S1.** Evidence-based list of cervical cancer risk factors and symptoms [27].

Symptoms
Vaginal bleeding between menstrual periods
Persistent lower back pain
Persistent smelly vaginal discharge
Discomfort or pain during sex
Menstrual periods that are longer or heavier than usual
Vaginal bleeding after menopause
Persistent lower abdominal/pelvic pain
Vaginal bleeding during or after sex
Blood in urine or stools
Unexplained weight loss
Persistent diarrhoea
**Risk factors**
Getting a sexually transmitted infection called the HPV
HIV/AIDs
Being infected with other sexually transmitted diseases (other than HIV or HPV)
Using birth control pills/family planning for more than 5 years
Having unprotected sex
Smoking any cigarettes at all
Having a sexual partner who is not circumcised
Having sex at a young age
Giving birth to three or more children
Having many sexual partners
Not going for regular screening/testing for cervical cancer

**Table S2.** Cervical cancer symptoms, risk factors and lay beliefs mentioned by women in this study.

	Overall *N* = 501 *n* (%)	SA *N* = 285 *n* (%)	Zimbabwe *N* = 216 *n* (%)
Symptoms			
Intermenstrual bleeding	21 (4.2)	2 (0.7)	19 (8.8)
Lower back pain	41 (8.2)	3 (1.1)	38 (17.6)
Vaginal discharge or smelly vaginal discharge	129 (25.7)	25 (8.8)	104 (48.1)
Discomfort/pain during sex	9 (1.8)	2 (0.7)	7 (3.2)
Longer or heavier menstrual periods	6 (1.2)	3 (1.1)	3 (1.4)
Post-menopausal bleeding	33 (6.6)	2 (0.7)	31 (14.4)
Vaginal or Lower abdominal/pelvic pain	99 (19.8)	29 (10.2)	70 (32.4)
Vaginal bleeding during or after sex	13 (2.6)	6 (2.1)	7 (3.2)
Unexplained weight loss	7 (1.4)	3 (1.1)	4 (1.9)
Blood in urine or stool	1 (0.2)	0 (0.0)	1 (0.5)
Persistent diarrhoea	0 (0.0)	0 (0.0)	0 (0.0)
Non-specific bleeding	149 (29.7)	49 (17.2)	100 (46.3)
Aggregated symptoms recall score			
Zero symptoms recalled	272 (54.3)	230 (80.7)	42 (19.4)
One symptom recalled	127 (25.3)	40 (14.0)	87 (40.3)
Two symptoms recalled	78 (15.6)	11 (3.9)	67 (31.0)
Three symptoms recalled	20 (4.0)	3 (1.1)	17 (7.9)
Four symptoms recalled	4 (0.8)	1 (0.4)	3 (1.4)
Five or more symptoms recalled	0 (0.0)	0 (0.0)	0 (0.0)
Risk factors			
HPV infection	4 (0.8)	2 (0.7)	2 (0.9)
HIV/AIDs	17 (3.4)	1 (0.4)	16 (7.4)
Sexually transmitted diseases	11 (2.2)	1 (0.4)	10 (4.7)
Prolonged birth control use	3 (0.6)	2 (0.7)	1 (0.5)
Unprotected sex	17 (3.4)	5 (1.8)	12 (5.6)
Smoking cigarettes	13 (2.6)	13 (4.6)	0 (0.0)
Sexual onset at a young age	3 (0.6)	1 (0.4)	2 (0.9)
Having many sexual partners or partner having many sexual partners	40 (8.0)	4 (1.4)	36 (16.7)
Not going for regular screening	7 (1.4)	5 (1.8)	2 (0.9)
Sexual partner not circumcised	0 (0.0)	0 (0.0)	0 (0.0)
Giving birth to three or more children	0 (0.0)	0 (0.0)	0 (0.0)
Aggregated risk factors recall score			
Zero risk factors recalled	408 (81.4)	260 (91.2)	148 (68.5)
One risk factor recalled	72 (14.4)	17 (6.0)	55 (25.5)
Two risk factors recalled	20 (4.0)	7 (2.5)	13 (6.0)
Three risk factors recalled	1 (0.2)	1 (0.4)	0 (0.0)
Four or more risk factors recalled	0 (0.0)	0 (0.0)	0 (0.0)
Risk factor lay beliefs			
Family history	14 (2.8)	4 (1.4)	10 (4.7)
Menopause	5 (1.0)	0 (0.0)	5 (2.3)
ARVs	3 (0.6)	0 (0.0)	3 (1.4)
Stress	3 (0.6)	0 (0.0)	3 (1.4)
Inserting herbs, creams or objects into vagina	43 (8.6)	0 (0.0)	43 (20.0)
Poor personal hygiene	4 (0.8)	2 (0.7)	2 (0.9)
Witchcraft/bewitched/evil spirits	13 (2.6)	0 (0.0)	13 (6.0)
Lifestyle related	9 (1.8)	6 (2.1)	3 (1.4)
General herbs use	4 (0.8)	0 (0.0)	4 (1.9)
Other*	22 (4.4)	2 (0.7)	20 (9.3)
At least one risk lay belief	90 (18.0)	11 (3.9)	79 (36.6)

* Includes risk lay beliefs that were mentioned by fewer than three participants

**Table S3.** Univariate regression analysis of symptom awareness for ea ch country.

	SA	Zimbabwe
Characteristics	Crude OR (95% CI)	Crude OR (95% CI)
Age, (years)		
18–34	Ref	Ref
35–44	1.01 (0.36; 3.12)	1.33 (0.18; 6.87)
45–54	0.70 (0.25; 2.16)	1.21 (0.17; 5.85)
>55	**0.28 (0.09; 0.93)**	1.67 (0.23; 8.14)
Relationship		
Married/living with partner	Ref	Ref
Single	0.79 (0.40; 1.55)	0.62 (0.19; 2.47)
Separated/divorced/widowed	0.48 (0.21; 1.03)	1.14 (0.56; 2.33)
Education		
Less than secondary school	Ref	Ref
Secondary school complete	**3.52 (1.78; 6.90)**	1.09 (0.53; 2.31)
Employment		
Unemployed	Ref	Ref
Employed	1.22 (0.61; 2.34)	1.00 (0.49; 2.14)
Expenditure index		
First quintile (poorest)	Ref	Ref
Second quintile	1.51 (0.61; 3.90)	**0.20 (0.05; 0.60)**
Third quintile (middle)	**2.68 (1.09; 6.92)**	0.44 (0.11; 1.58)
Fourth quintile	1.43 (0.47; 4.15)	**0.29 (0.07; 0.95)**
Fifth quintile	**3.19 (1.15; 9.01)**	0.53 (0.12; 2.15)
Self-reported living context		
Rural	Ref	Ref
Urban	**3.96 (2.02; 8.12)**	1.04 (0.52; 2.06)
Peri-urban	**5.40 (2.06; 13.98)**	2.82 (0.50; 53.22)
Known family member or friend with cancer		
No	Ref	Ref
Yes	**2.20 (1.21; 4.02)**	0.75 (0.38; 1.54)
Self-reported previous TB		
No	Ref	Ref
Yes	0.83 (0.42; 1.58)	1.45 (0.52; 5.15)
Self-reported hypertension		
No	Ref	Ref
Yes	0.88 (0.46; 1.64)	1.1 (0.55; 2.26)
Self-reported diabetes		
No	Ref	Ref
Yes	1.44 (0.54; 3.43)	1.46 (0.24; 28.06)
Self-reported cardiac disease		
No	Ref	Ref
Yes	1.31 (0.36; 3.88)	0.23 (0.03; 1.99)
Self-reported HIV		
No	Ref	Ref
Yes	0.82 (0.46; 1.49)	0.94 (0.47; 1.86)
Self-reported previous COVID		
No	Ref	Ref
Yes	2.77 (0.81; 8.68)	1.48 (0.38; 9.76)
Self-reported history of any chronic disease*		
No	Ref	Ref
Yes	0.69 (0.36; 1.37)	1.36 (0.60; 2.91)
Self-reported previous screening cervical cancer		
No	Ref	Ref
Yes	1.02 (0.36; 3.65)	0.90 (0.36; 2.03)

OR – Odds ratio

CI – confidence interval

* Chronic disease include: Hypertension, diabetes, HIV, cardiac disease

Bold OR & CI – variable statistically significant

**Table S4.** Univariate regression analysis of risk factor awareness in each country.

	SA Crude OR (95% CI)	Zimbabwe Crude OR (95% CI)
Characteristic		
Age, (years)		
18–34	Ref	Ref
35–44	1.43 (0.34; 9.79)	2.00 (0.41; 14.59)
45–54	1.01 (0.23; 7.06)	1.83 (0.39; 13.05)
>55	0.56 (0.11; 4.07)	0.82 (0.17; 5.91)
Relationship		
Married/living with partner	Ref	Ref
Single	0.58 (0.22; 1.46)	1.29 (0.39; 4.00)
Separated/divorced/widowed	0.44 (0.14; 1.25)	0.59 (0.32; 1.07)
Education		
Less than secondary school	Ref	Ref
Secondary school completed	**4.59 (1.91; 10.86)**	**1.94 (1.06; 3.54)**
Employment		
Unemployed	Ref	Ref
Employed	1.87 (0.76; 4.38)	**2.87 (1.56; 5.30)**
Expenditure index		
First quintile (poorest)	Ref	Ref
Second quintile	1.52 (0.42; 6.17)	0.74 (0.29; 1.86)
Third quintile (middle)	1.74 (0.44; 7.33)	1.25 (0.50; 3.15)
Fourth quintile	1.82 (0.41; 8.09)	1.83 (0.76; 4.51)
Fifth quintile (richest)	**3.94 (1.05; 16.47)**	1.88 (0.74; 4.85)
Self-report living context		
Rural	Ref	Ref
Urban	**3.32 (1.26; 9.73)**	1.62 (0.89; 2.99)
Peri-urban	**6.55 (1.90; 22.74)**	1.44 (0.36; 5.01)
Known family member or friend with cancer		
No	Ref	Ref
Yes	1.33 (0.56; 3.04)	1.71 (0.94; 3.11)
Self-reported previous TB		
No	Ref	Ref
Yes	0.55 (0.18; 1.41)	1.59 (0.68; 3.62)
Self-reported hypertension		
No	Ref	Ref
Yes	**0.36 (0.10; 0.97)**	0.66 (0.35; 1.20)
Self-reported diabetes		
No	Ref	Ref
Yes	0.36 (0.02; 1.80)	0.87 (0.12; 4.14)
Self-reported HIV		
No	Ref	Ref
Yes	0.47 (0.19; 1.08)	**3.01 (1.63; 5.76)**
Self-reported previous covid		
No	Ref	Ref
Yes	1.97 (0.29; 7.92)	1.69 (0.54; 5.07)
Self-reported cardiac disease		
No	Ref	Ref
Yes	1.42 (0.22; 5.47)	0.00
Self-reported history of any chronic disease*		
No	Ref	Ref
Yes	**0.16 (0.07; 0.38)**	1.93 (0.93; 4.36)
Self-reported previous cervical cancer screening		
No	Ref	Ref
Yes	0.91 (0.24; 5.91)	**2.46 (1.12; 5.99)**

OR – Odds ratio

CI – confidence interval

* Chronic disease include: Hypertension, diabetes, HIV, cardiac disease

Bold OR & CI – variable is statistically significant
